# Pre-clinical evidence of PIM kinase inhibitor activity in BCR-ABL1 unmutated and mutated Philadelphia chromosome-positive (Ph+) leukemias

**DOI:** 10.18632/oncotarget.5091

**Published:** 2015-09-07

**Authors:** Dany A. Curi, Elspeth M. Beauchamp, Gavin T. Blyth, Ahmet Dirim Arslan, Nicholas J. Donato, Francis J. Giles, Jessica K. Altman, Leonidas C. Platanias

**Affiliations:** ^1^ Robert H. Lurie Comprehensive Cancer Center of Northwestern University, Chicago, IL, USA; ^2^ Division of Hematology-Oncology, Ann & Robert H. Lurie Children's Hospital of Chicago, Northwestern University, Chicago, IL, USA; ^3^ Division of Hematology-Oncology, Department of Medicine, Feinberg School of Medicine, Northwestern University, Chicago, IL, USA; ^4^ Division of Hematology-Oncology, Department of Medicine, Jesse Brown VA Medical Center, Chicago, IL, USA; ^5^ Department of Pharmacology, University of Michigan School of Medicine, Ann Arbor, MI, USA

**Keywords:** CML, PIM kinase, mTOR signaling, philadelphia chromosome-positive leukemia

## Abstract

We investigated the efficacy of targeting the PIM kinase pathway in Philadelphia chromosome-positive (Ph+) leukemias. We provide evidence that inhibition of PIM, with the pan-PIM inhibitor SGI-1776, results in suppression of classic PIM effectors and also elements of the mTOR pathway, suggesting interplay between PIM and mTOR signals. Our data demonstrate that PIM inhibition enhances the effects of imatinib mesylate on Ph+ leukemia cells. We also found that PIM inhibition results in suppression of leukemic cell proliferation and induction of apoptosis of Ph+ leukemia cells, including those resistant to imatinib mesylate. Importantly, inhibition of PIM results in enhanced suppression of primary leukemic progenitors from patients with CML. Altogether these findings suggest that pharmacological PIM targeting may provide a unique therapeutic approach for the treatment of Ph+ leukemias.

## INTRODUCTION

Chronic myelogenous leukemia (CML) evolves from abnormal hematopoietic stem cells of myeloid origin. Its signature characteristic is the genetic translocation, t(9;22) (q34;q11) BCR-ABL1, also known as the Philadelphia chromosome (Ph+) [1, 2]. This abnormality is found in about 90 to 95% of CML, approximately 25% of adult acute lymphoblastic leukemia (ALL), and 3 to 4% of cases of pediatric ALL [1, 2]. The treatment of CML with tyrosine kinase inhibitors (TKIs) changed the way we approach the management of Ph (+) leukemias, and heralded a new era of clinical oncology with implications in many different fields. However, despite success with TKIs, some patients with early phase CML and the great majority of those with advanced disease develop resistance and/or intolerance to therapy, and represent a significant clinical challenge. Notably, over 90 mutations have been already identified in the BCR-ABL1 kinase domain [3, 4] and it is possible that additional ones will be identified with time. Options for patients with CML in whom multiple TKIs have failed include recently FDA approved drugs ponatinib and omacetaxine mepesuccinate [5–8]. However, significant adverse effects have been reported with these latter agents, and a significant number of patients treated with ponatinib have developed severe thrombosis and/or vascular occlusive disease [5, 9]. These difficulties in the management of patients with refractory Ph+ leukemias underscore the need to develop novel agents and unique therapeutic approaches to overcome resistance to BCR-ABL1 TKIs.

One pathway that has attracted attention for anticancer drug development is the proviral insertion site in Moloney murine leukemia virus (PIM) kinase pathway [10, 11]. To date, three PIM kinases (PIM1, PIM2, and PIM3) have been identified, and are characterized by substantial amino acid homology, as well as increased protein expression in different malignancies [10, 11]. Increased PIM kinase expression has been reported in prostate cancer, acute myeloid leukemia (AML), chronic lymphoblastic leukemia (CLL), and non-Hodgkin's lymphoma [10–12]. In addition, previous studies have shown that there may be functional redundancies of the different *PIM* genes, as *PIM1−/−* mice do not have a significant phenotype [13] compared with *PIM1/2/3 −/−* mice [14]. Furthermore, loss of *PIM1* in a lymphoma mouse model resulted in upregulation of *PIM2* [15]. Thus, treatment of malignancies using agents with pan-PIM inhibitory properties may be important in hindering potential compensatory effects and optimizing responses.

Although not yet well understood, the PIM kinase pathway is involved in the regulation or transmission of many signals that lead to leukemogenesis. For example, PIM-mediated phosphorylation of histone 3 at serine 10 increases *MYC*-dependent transcription of genes such as *MCL-1* [12, 16]. Cell cycling is increased by PIM kinases phosphorylating cyclin dependent kinase inhibitors (p21 and p27) and phosphatases (cdc25a and cdc25c) [17–20]. PIM kinases promote cell survival by phosphorylation of BAD at Ser112 [21–23]. Another mechanism by which PIM kinases are involved in leukemogenesis appears to involve cross-talk with the mammalian target of rapamycin (mTOR) pathway. PIM1 has been shown to directly phosphorylate PRAS40 at Thr246 [24], while there is evidence that PIM2 is upstream of mTORC1 and regulates its activity by phosphorylating TSC2 [25].

In the present study, we sought to test the efficacy of PIM inhibition alone or in combination with imatinib mesylate on Ph+ leukemia cells. Our data shows that inhibition of PIM, with the pan-PIM inhibitor SGI-1776, results in suppression of the mTOR pathway as well as other downstream effectors. We also found reduced leukemic cell proliferation, induction of apoptosis, and inhibition of colony formation in Ph+ cell lines including those resistant to imatinib. In imatinib-sensitive cell lines, an enhanced effect was seen when combining inhibition of PIM with imatinib. Moreover, we establish that PIM inhibition results in suppressive effects on primary leukemic progenitors from CML patients, further suggesting a potential role for PIM targeting as a novel therapeutic approach for Ph+ leukemias.

## RESULTS

**Figure 1:**
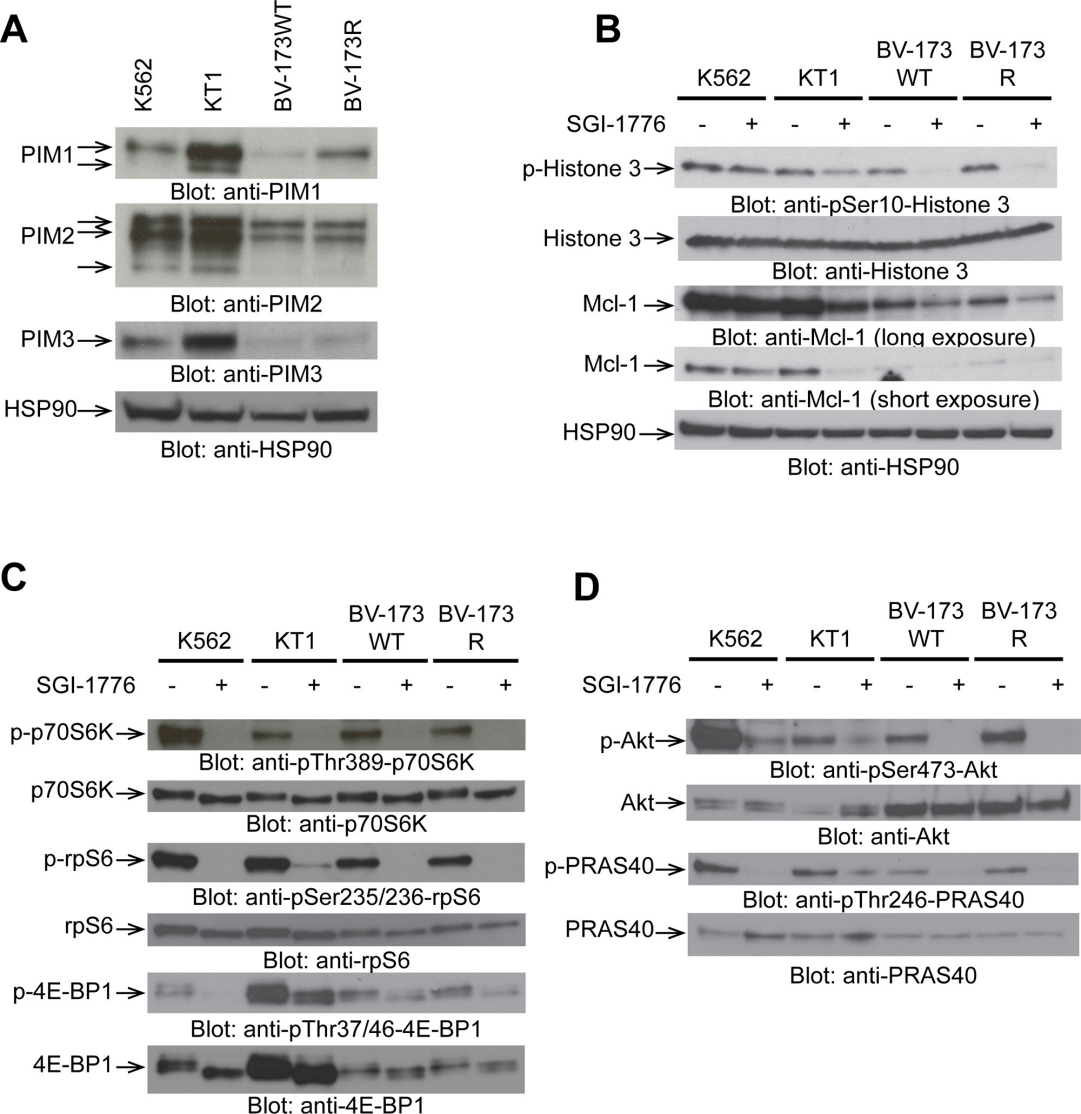
Expression of PIM isoforms in BCR-ABL transformed cells and inhibitory effects of SGI-1776 on PIM effectors **A.** Total cell lysates from K562, KT1, BV173WT, and BV173R cell lines were resolved by SDS-PAGE and immunoblotted with the indicated antibodies. **B, C, D.** K562, KT1, BV173WT, and BV173R cell lines were treated with SGI-1776 (10 μmol/L) for 2 hours, and total lysates were resolved by SDS-PAGE and immunoblotted with the indicated antibodies. The immunoblots with antibodies against the phosphorylated forms of the proteins or against the total proteins were from lysates from the same experiments analyzed in parallel by SDS-PAGE. In the case of PRAS40, after immunoblotting with the anti-phospho-PRAS40 antibody, the same blot was stripped and re-blotted using anti-PRAS40 antibody.

In initial experiments, we assessed the expression of all 3 PIM kinases in K562, KT1, BV173, and BV173R cell lines, by immunoblotting. As shown in Figure 1A, different patterns of expression of PIM isoforms were noticeable in the different lines. PIM1 was expressed in all lines (Figure 1A). KT1 cells expressed both isoforms of PIM1, 34 and 44 kDa, [10, 11] while the T315I kinase domain mutation cell line, BV173R [26], exhibited higher levels of expression of PIM1 compared to wild-type BV173 cells (Figure 1A). All 3 isoforms of PIM2 (34, 37, and 40 kDa isoforms) were expressed in K562 and KT1 cells, while BV173 and BV173R cells mainly expressed 2 isoforms; 37 and 40 kDa [10, 11] (Figure 1A). PIM3 was mainly expressed in K562 and KT1 cells, and to a lesser extent in BV-173 cells (Figure 1 A). Taken together, these findings suggested that pan-PIM inhibition would be important for induction of antileukemic responses, as PIM kinases have functional redundancies and the ability to compensate for each other [13–15].

We subsequently examined the effects of SGI-1776 on downstream components of the PIM kinase pathway. Treatment with SGI-1776 inhibited the phosphorylation of histone 3 on serine 10 and as well as Mcl-1 expression (Figure 1B). When the effects of SGI-1776 on components of the mTOR pathway were assessed, we found that the phosphorylation of several mTOR effectors was inhibited in the different cell lines. Specifically, phosphorylation of p70S6 kinase at Thr389, ribosomal protein S6 ser235/236, 4E-BP1 at Thr 37/46, (Figure 1C), as well as phosphorylation of AKT on Ser473 and PRAS40 on Thr246 (Figure 1D) were significantly inhibited by SGI-1776.

**Figure 2:**
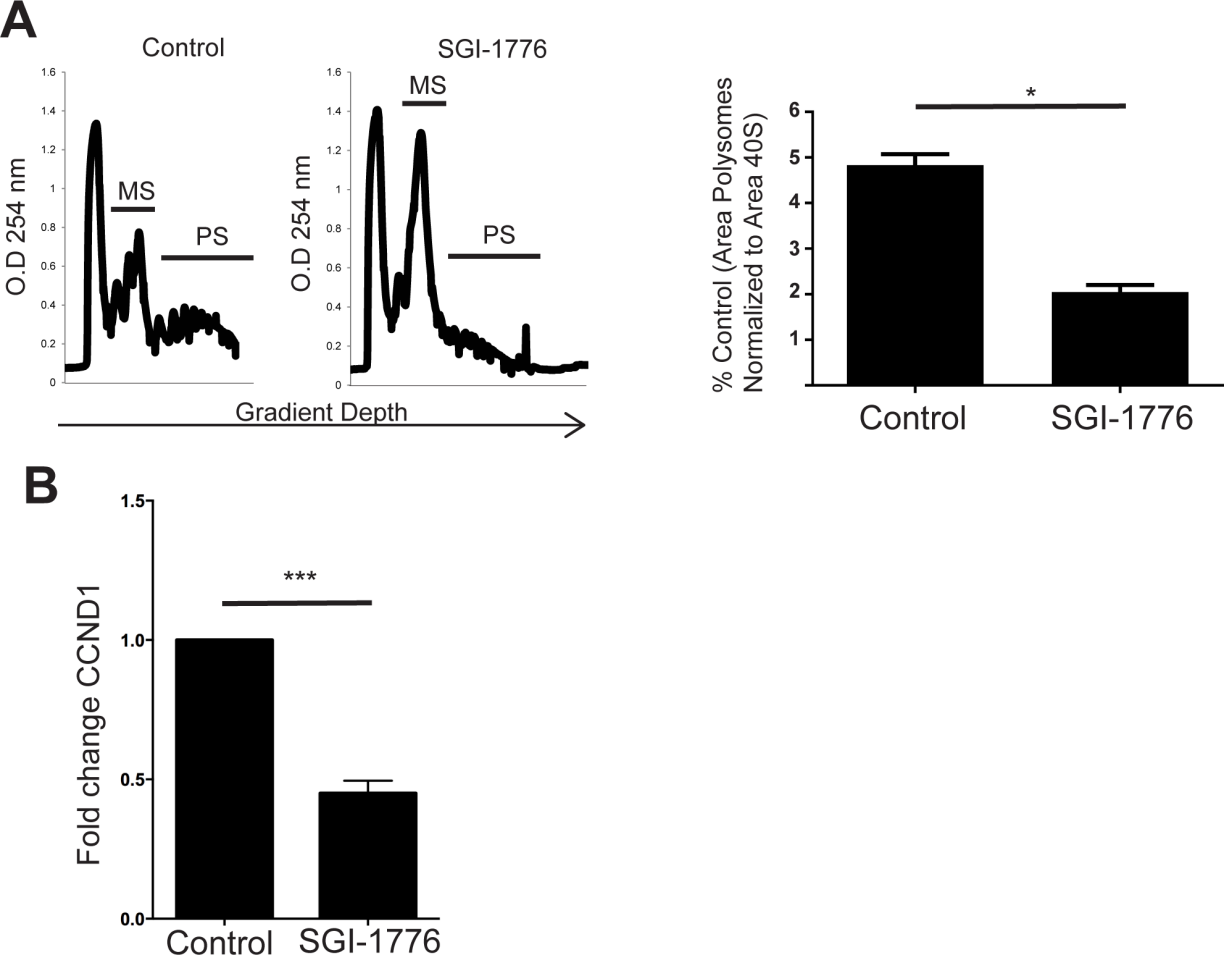
Pharmacological inhibition of PIM1 suppresses polysomal mRNA expression and mRNA translation of CCND1 in Ph+ leukemia cells **A.** *(Left panel)* Representative polysome profiles of KT1 cells. KT1 cells were treated with control (DMSO) or SGI-1776 (10 μmol/L) for two hours and lysates were separated on 10–50% sucrose gradients. The gradients were subjected to ultracentrifugation, and fractions were collected by continuous monitoring of optical density (OD) at 254 nm. The OD 254 nm is shown as a function of gradient depth for control cells or cells treated with SGI-1776. *(Right panel)* The area under the polysome peaks and polysome (PS) + monosome (MS) peaks was quantified for control and SGI-1776 by using Image J software. The ratio of area under the polysome and polysome plus monosomal peaks was calculated for SGI-1776 and is represented as percent control (DMSO). Shown are the means + SE of 3 independent experiments. *p < 0.05 using a paired *t*-test. **B.** The polysomal fractions were pooled and the total RNA was isolated. Quantitative real-time RT-PCR assay to determine the cyclin D1 (CCND1) mRNA expression in polysomal fractions was carried out using GAPDH as a control. Data are expressed as fold change as compared with DMSO control-treated samples and represent means + SE of 3 independent experiments. ***, *p* < 0.001 using a paired *t*-test.

The potent inhibitory effects of SGI-1776 on PIM effectors and elements of the mTOR pathway suggested potential cross-talk between PIM- and mTOR-mediated signals in BCR-ABL1 transformed cells. This led us to determine whether there is inhibition at the level of cap-dependent mRNA translation. To assess whether PIM inhibition resulted in inhibitory effects on cap-dependent mRNA translation, polysomal fractionation analysis was carried out. SGI-1776 treatment of KT1 cells treatment resulted in marked suppression of polysomal peaks (Figure 2A, *left panel*). Furthermore, the area under the curve (comparison of the polysomal profiles) indicated over two-fold reduction in polysomal fractions (Figure 2A, *right panel)*. We also examined whether SGI-1776 modulates polysomal mRNA levels for cyclin D1 (*CCND1*), a gene encoding for a protein that promotes cell proliferation and is highly expressed in different malignancies [27]. Real-time qRT-PCR assays demonstrated a significant SGI-1776-dependent reduction of polysome-associated *CCND1* transcript levels in KT1 cells (Figure 2B), consistent with inhibitory effects on *CCND1* mRNA translation.

**Figure 3:**
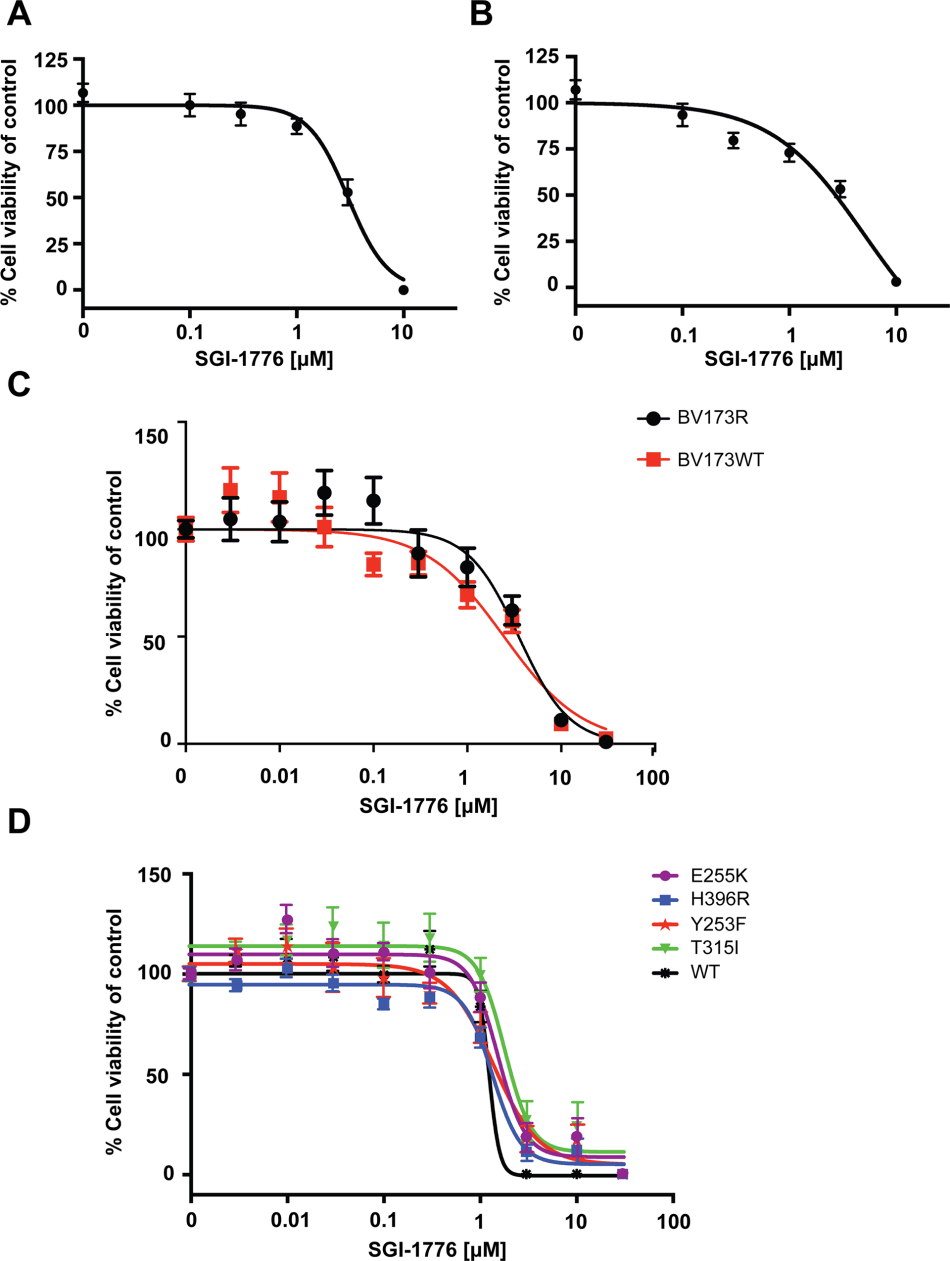
Suppressive effects of PIM inhibition on proliferation of Ph+ leukemia cell lines K562 **A.** and KT1 **B.** cells were plated in 96-well plates and treated with increasing concentrations of SGI-1776 for four days. Viability was assessed using a WST-1 assay. Data are expressed as a percentage of DMSO control-treated cells. Shown are the means ± SE of six independent experiments. **C.** BV173 and BV173R cells were plated in 96-well plates and treated with increasing concentrations of SGI-1776 for four days and viability was measured by using a WST-1 assay. Data are expressed as a percentage of DMSO control-treated cells. Shown are the means ± SE of four independent experiments. **D.** Ba/F3 p210WT, Ba/F3 p210E255K, Ba/F3 p210H396R, Ba/F3 p210Y253F, and Ba/F3 p210T315I cells were plated in triplicate in 96-well plates and treated with increasing concentrations of SGI-1776 for four days. Viability was measured using a WST-1 assay. Data are expressed as a percentage of DMSO control-treated cells. Shown are the means ± SE of three independent experiments.

PIM kinases promote cell cycling, proliferation, and cell survival [10–12]. We therefore determined whether PIM inhibition results in growth inhibitory/cytotoxic effects in the Ph+ leukemia cell lines. In WST-1 viability assays, we found that the IC50 values of inhibition by treatment with SGI-1776 were 3.5 μmol/L and 5 μmol/L for K562 and KT1 cells, respectively (Figures 3A and 3B). We also examined the effects of the inhibitor on BV173 cells and BV173 cells harboring the T315I mutation (BV173R). The IC50 value for wild-type BV173 was 2.5 μmol/L versus 3.5 μmol/L for BV173R (Figure 3C). The IC50 values were comparable, suggesting that PIM kinase inhibitors have activity against T315I-BCR-ABL1 expressing cells *in vitro*. In addition to the T315I kinase domain mutation, there are other mutations that can cause imatinib-resistance [3, 4]. We therefore further assessed the effects of SGI-1776 on cell viability of Ba/F3 cells expressing mutants for 3 other commonly seen mutations, in addition to T315I, and compared their IC50 values to their wild-type counterpart. The IC50 values for Ba/F3 p210 kinase domain mutations of E255K, H396R, Y253F, and T315I were 1.5 μmol/L, 1.3 μmol/L, 1.3 μmol/L, and 1.8 μmol/L, respectively (Figure 3D). These IC50 values were comparable with the WT IC50 value of 1.2 μmol/L. All cell lines were sensitive to SGI-1776, suggesting that treatment with SGI-1776 is effective in reducing cell viability in TKI-resistant cells expressing distinct BCR-ABL mutations.

**Figure 4:**
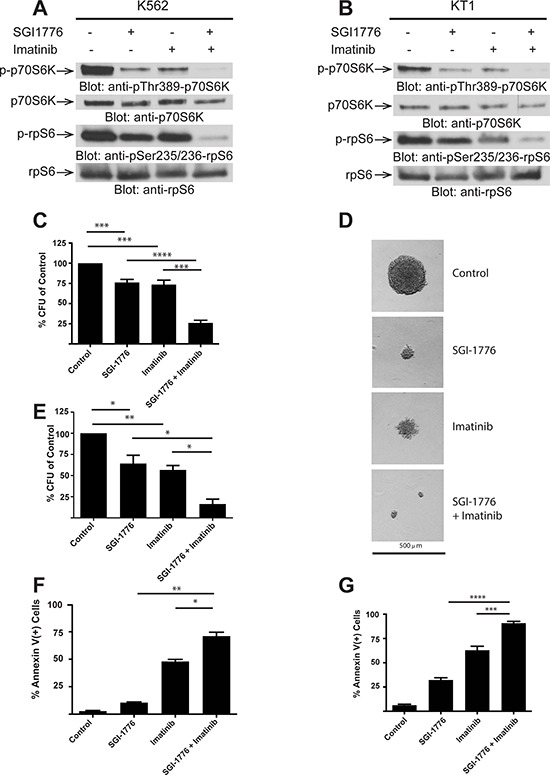
SGI-1776 enhances the suppressive effects of imatinib mesylate on BCR-ABL expressing cells **A.** K562 cells were treated with control (DMSO), SGI-1776 (3 μmol/L) or imatinib (0.5 μmol/L) alone or in combination for two hours, and equal amounts of cell lysates were resolved by SDS-PAGE and immunoblotted with the indicated antibodies. The immunoblots with antibodies against the phosphorylated forms of the proteins or against the total proteins were from lysates from the same experiments analyzed in parallel by SDS-PAGE.**B.** KT1 cells were treated with control (DMSO), SGI-1776 (3 μmol/L) or imatinib (1 μmol/L) alone or in combination for two hours, and equal amounts of cell lysates were resolved by SDS-PAGE and immunoblotted with the indicated antibodies. The immunoblots with antibodies against the phosphorylated forms of the proteins or against the total proteins were from lysates from the same experiments analyzed in parallel by SDS-PAGE. **C.** K562 cells were plated in methylcellulose in the presence of control (DMSO), SGI-1776 (10 μmol/L) or imatinib (0.5 μmol/L) or in combination for approximately seven days. Leukemic progenitor colony formation (CFU-L) was assessed in clonogenic assays in methylcellulose. Data are expressed as a percentage of DMSO control-treated cells. Shown are the means + SE of six independent experiments. ***p < 0.001, ****p < 0.0001 using a paired t-test.**D.** Representative colonies treated with control (DMSO), SGI-1776, imatinib, and SGI-1776 plus imatinib, from the experiments described in panel C is shown (x 10 magnification). **E.** LAMA-84 cells were plated in methylcellulose in the presence of control (DMSO), SGI-1776 (3 μmol/L) or imatinib (0.5 μmol/L) alone or in combination. Leukemic progenitor colony formation (CFU-L) was assessed in clonogenic assays in methylcellulose. Data are expressed as a percentage of DMSO control-treated cells. Shown are the means + SE of five independent experiments. *p < 0.05, **p < 0.01 using a paired t-test. **F.** K562 cells were treated with control (DMSO), SGI-1776 (3 μmol/L) or imatinib (0.5 μmol/L) alone or in combination for 24 hours. The percentage of apoptosis was determined by flow cytometry using Annexin V and DAPI stain. Shown are the means + SE of three independent experiments. *p < 0.05, **p < 0.01 using a paired t-test **G.** KT1 cells were treated with control (DMSO), SGI-1776 (3 μmol/L) or imatinib (1 μmol/L) alone or in combination for 24 hours. The percentage of apoptosis was determined by flow cytometry using Annexin V and DAPI stain. Shown are the means + SE of three independent experiments. ***p < 0.001, ****p < 0.0001 using a paired t-test.

We next sought to determine whether the combination of SGI-1776 with imatinib mesylate exhibits more potent effects than each agent alone. The combination of imatinib mesylate with low concentrations of SGI-1776 resulted in more potent suppression of the phosphorylation of the downstream mTOR substrates, ribosomal protein S6 at ser235/236 and p70 S6 kinase at Thr389 (Figure 4A and 4B). SGI-1776 also suppressed K562- and LAMA-84-derived leukemic progenitor colony formation (Figure 4C and 4E), while there was an enhanced inhibition of colony formation by combining SGI-1776 with imatinib mesylate (Figure 4C and 4E). Interestingly, there was noticeable reduction in leukemic progenitor colony size by SGI-1776 treatment of cells (Figure 4D). When the effects of SGI-1776 and imatinib mesylate on apoptosis were examined, we found significant induction of apoptosis in K562 cells by treatment with either SGI-1776 or imatinib mesylate, but such apoptotic cell death was further enhanced by the combination of the two agents (Figure 4F and 4G).

**Figure 5:**
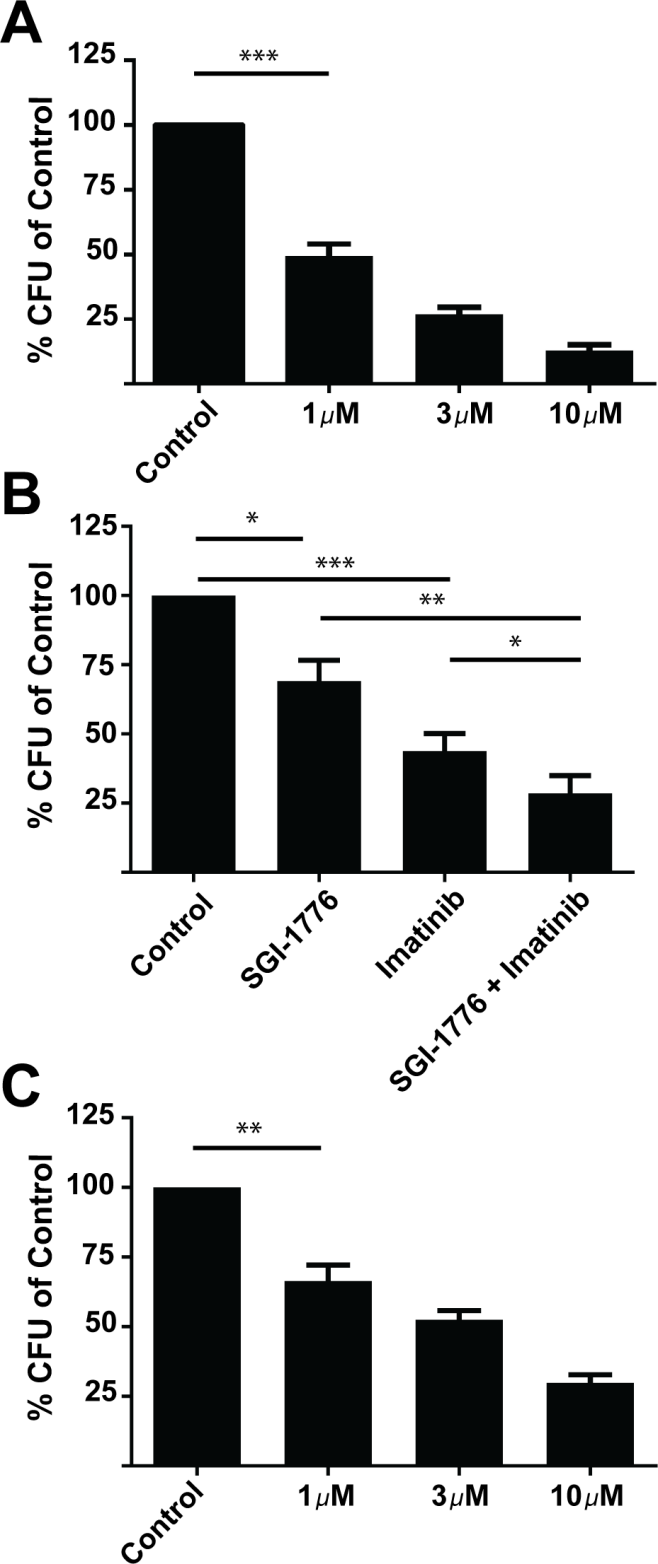
Effects of PIM kinase inhibition on primary leukemic progenitors from CML patients **A.** Primary mononuclear cells derived from CML patient samples were plated in methylcellulose in the presence of control (DMSO) or SGI-1776 at the indicated concentrations for approximately fourteen days. Leukemic progenitor colony formation (CFU-GM) was assessed in clonogenic assays in methylcellulose. Data are expressed as a percentage of DMSO control-treated cells. Shown are the means + SE of five experiments performed with samples from five different patients. ***p < 0.001 using a paired *t*-test. **B.** Primary mononuclear cells derived from CML patient samples were plated in methylcellulose in presence of control (DMSO), SGI-1776 (0.5 μmol/L) or imatinib (0.5 μmol/L) alone or in combination for approximately fourteen days, as indicated. Leukemic progenitor colony formation (CFU-GM) was assessed in clonogenic assays in methylcellulose. Data are expressed as a percentage of DMSO control-treated cells. Shown are the means + SE of five experiments performed with samples from five different patients. *p < 0.05, **p < 0.01, ***p < 0.001 using a paired *t*-test **C.** Primary mononuclear cells derived from CML patient samples were plated in methylcellulose in the presence of control (DMSO) or AZD-1208 at the indicated doses for approximately fourteen days. Leukemic progenitor colony formation (CFU-GM) was assessed in clonogenic assays in methylcellulose. Data are expressed as a percentage of DMSO control-treated cells. Shown are the means + SE of four experiments performed with samples from four different patients. **p < 0.01 using a paired *t*-test.

In further studies, we examined the antileukemic effects of two pan-PIM inhibitors, SGI-1776 and AZD-1208, on primitive leukemic progenitors from patients with CML. There was significant reduction of primary leukemic CFU-GM progenitor colony formation by either SGI-1776 (1 μmol/L) (Figure 5A) or AZD-1208 (1 μmol/L) (Figure 5C). When lower concentrations of SGI-1776 (0.5 μmol/L) alone or in combination with imatinib (0.5 μmol/L) were used, there was a significant reduction in colony formation when comparing control with SGI-1776 or imatinib mesylate, and SGI-1776 plus imatinib mesylate in combination (Figure 5B). Importantly, an enhanced reduction in colony formation was seen at very low doses of SGI-1776 (0.5 μmol/L) when combined with imatinib mesylate (Figure 5B). Notably, SGI-1776 and AZD-1208 were extremely potent in suppressing colony formation in primary CML patient-derived leukemic progenitors at significantly lower doses when compared to K562 and LAMA-84 cell lines (Figure 5B).

## DISCUSSION

The PIM kinase pathway involves many signaling elements that play roles in the regulation of leukemogenesis [12, 28–31]. Because of their important roles in malignant cell proliferation and survival, PIM kinases are attractive therapeutic targets. It was previously shown that PIM1 was important for BCR-ABL1-mediated transformation [32]. Ph+ cell lines have been shown to have an increased half-life of PIM1 leading to an increase in PIM1 expression [33]. In addition, there is some evidence that RNAi-targeting of PIM1 and PIM2 impairs survival of hematopoietic cells transformed by oncogenic protein tyrosine kinases, including BCR-ABL1 [34]. In the present study, we demonstrate that pharmacological inhibition of PIM kinases blocks both classical PIM targets and elements of the mTOR pathway, suggesting interplay between PIM and mTOR signaling in the context of BCR-ABL1 transformation.

Both mTORC1 and mTORC2 have been shown to be activated in CML [26, 35]. Previous studies have also shown that PIM kinases activate mTORC1 signaling through phosphorylation and inhibition of the negative regulators of the pathway, TSC2 and PRAS40 [24, 25, 31]. The phosphorylation of these substrates was significantly inhibited by SGI-1776, supporting a regulatory involvement of PIM kinase activity on mTORC1 engagement, likely similar to what is observed in AML [31]. In our studies, the phosphorylation of AKT at Ser473 by mTORC2 is inhibited by SGI-1776 suggesting a potential regulatory effect of PIM kinases on mTORC2. The potential mechanism by which PIM kinases modulate mTORC2 is unknown at this time and should be the focus of future studies. Altogether, our data clearly establish that SGI-1776 significantly inhibits components of the mTOR pathway in Ph+ cell lines.

Importantly, our studies demonstrate that PIM kinase inhibition of mTORC1 signals results in negative regulatory effects on mRNA translation of genes encoding for mitogenic proteins in BCR-ABL transformed cells, as evidenced by the effects on leukemic polysomal profiles and the inhibition of mRNA translation of the *Cyclin D1* gene, providing a mechanism or generation of growth inhibitory responses by PIM inhibition. Moreover, our data establish that PIM activity plays an important role in survival of BCR-ABL1 transformed cells, as shown by the induction of leukemic cell apoptosis in response to PIM pharmacological targeting. A possible mechanism for the induction of apoptosis may involve modulating phosphorylation of histone H3 on serine 10 that leads to effects on MYC-dependent transcription [16]. Consistent with this hypothesis we have found that treatment of different Ph+ leukemia cell lines with the PIM inhibitor SGI-1776 results in inhibition of phosphorylation of histone H3 on Ser10. Taken together with previous studies that showed similar effects in AML [29] but not in CLL cell lines [28], our findings raise the possibility of specific function of the PIM pathway in myeloid cells. Consistent with an effect of SGI-1776 on MYC-dependent transcription we also see reduction in the level of protein expression of the MYC-dependent gene, MCL-1. MCL-1 has been shown to promote survival in BCR-ABL1 transformed cells [36, 37]. Notably, other studies have established PIM inhibition-dependent decrease in expression of MCL-1 [12, 28, 29].

Finally, we provide direct evidence that PIM inhibition results in potent suppression of primitive leukemic progenitors from patients with CML and enhances antileukemic responses to imatinib mesylate *in vitro.* Notably, pharmacological PIM inhibition appears to exhibit activity against T315I-BCR-ABL1-expressing cells, suggesting that targeting the PIM kinase pathway may provide an approach to overcome resistance to tyrosine kinase inhibitors in CML. Future studies to test PIM inhibitors in *in vivo* mouse models of CML or Ph+ ALL, especially imatinib resistant models, is warranted and may have important clinical-translational implications for the treatment of CML and Ph+ ALL.

## MATERIALS AND METHODS

### Cell lines and reagents

Ba/F3 p210 WT and kinase domain mutations including T315I, E255K, H396R, and Y253F were kindly provided by Dr. Brian J. Druker (Howard Hughes Medical Institute and Oregon Health & Science University, Portland, OR). K562, KT1, and Ba/F3 p210 cell lines were cultured in RPMI 1640 medium supplemented with 10% fetal bovine serum (FBS). BV173, BV173R [26] and LAMA-84 cells were cultured in RPMI 1640 medium supplemented with 20% FBS. The pan-PIM inhibitors SGI-1776 [28, 29] and AZD-1208 [31, 38] were purchased from Selleckchem (Houston, Texas). Imatinib mesylate was purchased from ChemiTek. All drug agents were dissolved in dimethyl sulfoxide (DMSO), and cells were treated with the indicated doses and times.

### Immunoblotting assays

Immunoblotting experiments were performed as previously described [39]. All antibodies were purchased from Cell Signaling except for GAPDH (Millipore), PIM1 (Santa Cruz Biotechnology), and HSP90 (Santa Cruz Biotechnology).

### WST-1 Cell viability assays

These assays were performed essentially as previously described [39]. Briefly, cells were plated in triplicate in 96 well plates and allowed to incubate for 4 days with increasing concentrations of SGI-1776. Cell viability was assessed by adding WST-1 (Roche) to each well according to the manufacturer's instructions and was measured using an Epoch Microplate Spectrophotometer (BioTek). IC50 curves were generated using Prism GraphPad 6.0.

### Clonogenic leukemic progenitor assays in methylcellulose

These assays were performed essentially as in our previous studies [26]. Peripheral blood or bone marrow samples were obtained from patients with CML after obtaining informed consent approved by the Institutional Review Board of Northwestern University. Mononuclear cells were isolated by Ficoll-Hypaque (Sigma Aldrich) sedimentation. To assess the effects of drugs on leukemic progenitor (CFU-L) colony formation, cells were then plated in methylcellulose (MethoCult™ H4534 Classic without EPO, Stem Cell Technologies).

### Polysomal profiling and fractionation

Briefly, cells were washed twice with Dulbecco's phosphate buffered saline (DPBS) with 100 μg/ml cycloheximide and then lysed in lysis buffer (0.5% Triton X 100, 0.5% sodium deoxycholate, 5 mM Tris, pH 7.5, 2.5 mM MgCl2, 1.5 mM KCl 100 μg/ml cycloheximide, 2 mM DTT, protease inhibitor and 1U/μl RNase inhibitor). Lysates were then centrifuged at 12000g for 5 minutes at 4°C and supernatants were collected and snap frozen in liquid nitrogen. To isolate ribosomal fractions, lysates were layered on a sucrose gradient of 5 to 50%. Samples were centrifuged at 4°C for 110 minutes at 35000 rpm in a Beckman SW41-Ti rotor. The absorbance was measured at 254 nm continuously in an ISCO density gradient fractionator with the following settings: pump speed, 0.80 ml/min; fraction size, 10 drops per fraction; chart speed, 300 cm per hour; sensitivity, 1; peak separator, off; noise filter, 0.5 seconds. A 5% sucrose solution was used to set the baseline in an UA-6 detector for all experiments. Polysomal RNA from the fractions were isolated using a Qiagen AllPrep RNA/Protein kit. RNA was reverse transcribed using oligodT primers and the Qiagen Omniscript RT PCR kit, according to the manufacturer's instructions. Quantitative real-time (RT) PCR was carried out by using validated Taqman FAM-labeled probes, and Taqman PCR master mix (Life Technologies) according to the manufacturer's instructions. The probes used were as follows: GAPDH, Hs99999905_m1; Cyclin D1, Hs00765553_m1. GAPDH was used for normalization as indicated, and results were plotted as a ratio of Cyclin D1/GAPDH.

### Assessment of apoptosis by flow cytometry

Briefly, either 1 or 2 × 10^5^ of K562 or KT1 cells were plated per well and treated for 24 hours with SGI-1776, imatinib mesylate, or SGI-1776 plus imatinib mesylate. Samples were stained with Annexin V and 4′-6-diamidino-2-phenylindol (DAPI)-DNA stain according to the manufacturer's instructions (BD Biosciences). Fluorescence was measured on stained cells using a LSRFortessa Analyzer (BD Biosciences). Data was analyzed using FlowJo software (Tree Star, Ashland, OR).

### Statistical analysis

All statistical analyses were performed using Prism GraphPad 6.0.
